# Therapeutic Effects of Bifidobacterium breve YH68 in Combination with Vancomycin and Metronidazole in a Primary Clostridioides difficile-Infected Mouse Model

**DOI:** 10.1128/spectrum.00672-22

**Published:** 2022-03-21

**Authors:** Jingpeng Yang, Lingtong Meng, Hong Yang

**Affiliations:** a School of Food Science and Pharmaceutical Engineering, Nanjing Normal University, Nanjing, China; b State Key Laboratory of Microbial Metabolism, School of Life Science & Biotechnology, Shanghai Jiao Tong University, Shanghai, China; Johns Hopkins Hospital

**Keywords:** *Bifidobacterium breve*, vancomycin, metronidazole, *Clostridioides difficile*, fecal microbiota, metabolome

## Abstract

Probiotics have been widely used to prevent primary Clostridioides difficile infection (pCDI); however, there are fewer studies on their therapeutic aspects for pCDI. In this study, high doses of Bifidobacterium breve YH68 were used alone or in combination with vancomycin (VAN) and metronidazole (MTR) to treat pCDI mice. Mouse feces were collected from preinfection, postinfection, and posttreatment stages. Subsequently, the C. difficile number and toxin level in feces were detected by plate count method and C. difficile toxin enzyme-linked immunosorbent assay (ELISA). Simultaneously, 16S rRNA amplicon sequencing and untargeted metabolomics were employed to explore the changing patterns and characteristic markers of fecal microbiota and metabolome. The results indicated that high doses of YH68 used alone or in combination with VAN and MTR were more effective than the combination of VAN and MTR for pCDI mice and improved their final survival rate. This probiotic strain and its combination with antibiotics reduced C. difficile numbers and toxin levels in the feces, downregulated proinflammatory cytokine levels in colon tissue, and alleviated cecum tissue hyperplasia. Meanwhile, the level of fecal microbiota diversity increased significantly in pCDI mice after treatment, with an increase in the relative abundance of *Bifidobacterium*, *Akkermansia*, *Oscillospira*, unidentified*_S24-7*, and *Ruminococcus*, and this process was accompanied by elevated levels of secondary bile acid, butyric acid, and gentamicin C1a and reduced levels of primary bile acid and indoles. Most notably, the combination of YH68 with VAN and MTR diminished the damaging effect of antibiotic treatment alone on the microbiota. Our findings suggested that high doses of YH68 used in combination with VAN and MTR have a better therapeutic effect on pCDI mice than the combination of VAN and MTR alone.

**IMPORTANCE** Many studies have focused on the preventive effects of probiotics against pCDI, but few studies have investigated in depth the therapeutic effects of probiotics, especially at the postinfection stage. We demonstrated that high doses of Bifidobacterium breve YH68 used alone or in combination with vancomycin (VAN) and metronidazole (MTR) exerted outstanding efficacy in the treatment of pCDI mice. This probiotic-antibiotic combination regimen has the potential to be a new option for the clinical treatment of pCDI.

## INTRODUCTION

Clostridioides difficile is a Gram-positive obligate anaerobic bacterium commonly found in the human and mammalian intestines (1). Infectious diseases triggered by these microbes are called C. difficile infections (CDI), which pose an enormous danger to public health and a heavy burden on the national treasury (2). Antibiotics such as metronidazole (MTR) and vancomycin (VAN) are the mainstream therapy for primary C. difficile infection (pCDI), although the recurrence rate is high (3). Repeated infections and antibiotic treatment can progress to severe recurrent CDI (rCDI), which is more difficult to treat (2). For these troublesome cases, fecal microbiota transplantation (FMT) is currently the preferred option because of the high cure and survival rates associated with this therapy (4). However, FMT still has some shortcomings, including the lack of uniform standards (especially for pediatric cases), high operational costs, and the potential risk factors of stool samples from donors (5). Therefore, for the intermediate process between pCDI and progressive development of severe rCDI, there is an urgent need to find a mild, safe, and widely accepted method of prevention or treatment to avoid further deterioration.

In recent years, probiotics have shown efficacy in preventing and alleviating gastrointestinal diseases, especially in some specific intestinal diseases, such as pCDI (6). Most studies have focused on the clinical preventive effects of probiotics on pCDI, examining whether people can avoid infection by consuming probiotics earlier (2, 7). Shen et al. found that probiotics used alone or in combination with antibiotics significantly prevented pCDI and reduced final infection rates (7). Theoretically, these results imply that there may also be probiotic strains capable of treating pCDI. However, a few studies have shown that probiotics do not seem to be significant or even effective in the treatment of pCDI, and the mechanism behind this is not yet clear (8). The actual effectiveness of probiotics is still a controversial topic. There is a consensus that the dynamics of gut microbial structure and changes in the levels of key metabolites play a central role in many infectious diseases of the gut (e.g., pCDI) (9). Thus, the study of the microbiota and metabolites is crucial to reveal the pathology or the recovery of intestinal homeostasis of pCDI. The pCDI mouse model can simulate the pathological process of clinical pCDI and has been used widely (10–12). De Wolfe et al. found a higher abundance of *Lachnospiraceae* in the fecal microbiota of mice with moderate CDI treated with continuous probiotics, and these probiotic strains brought the mouse model a delayed onset of pCDI and relapse (11), which is strong evidence that probiotics have therapeutic potential for pCDI. However, that work contains four different strains of probiotics that are ingested continuously during the pre- and postinfection stages, more like CDI prevention. It should be clear that the modes of action of probiotics in the prevention of pCDI and in the treatment of pCDI are different; the former means that the early intake of probiotics can prevent the invasion of C. difficile, while the latter means that the probiotics start to fight C. difficile only after it has successfully invaded and colonized the gut and triggered disruption of homeostasis (5).

To our knowledge, previous studies have focused on the prevention of pCDI (11–15), but few studies have involved the treatment; especially whether there are probiotic strains that can play a positive therapeutic role after the onset of pCDI is unknown. Bifidobacterium breve YH68 is a probiotic strain with outstanding antagonistic effects against C. difficile, including YH68 used alone or in combination with antibiotics *in vitro* (16–18). Here, we used YH68 alone or in combination with VAN and MTR to treat pCDI model mice to verify their actual therapeutic effects. Simultaneously, we investigated the characteristics and correlation of fecal microbiota and metabolites in pCDI mice treated with different therapeutic regimens and preliminarily explored the therapeutic mechanism of YH68 in combination with VAN and MTR.

## RESULTS

### Therapeutic effects of different treatments on pCDI mice.

**FIG 1:**
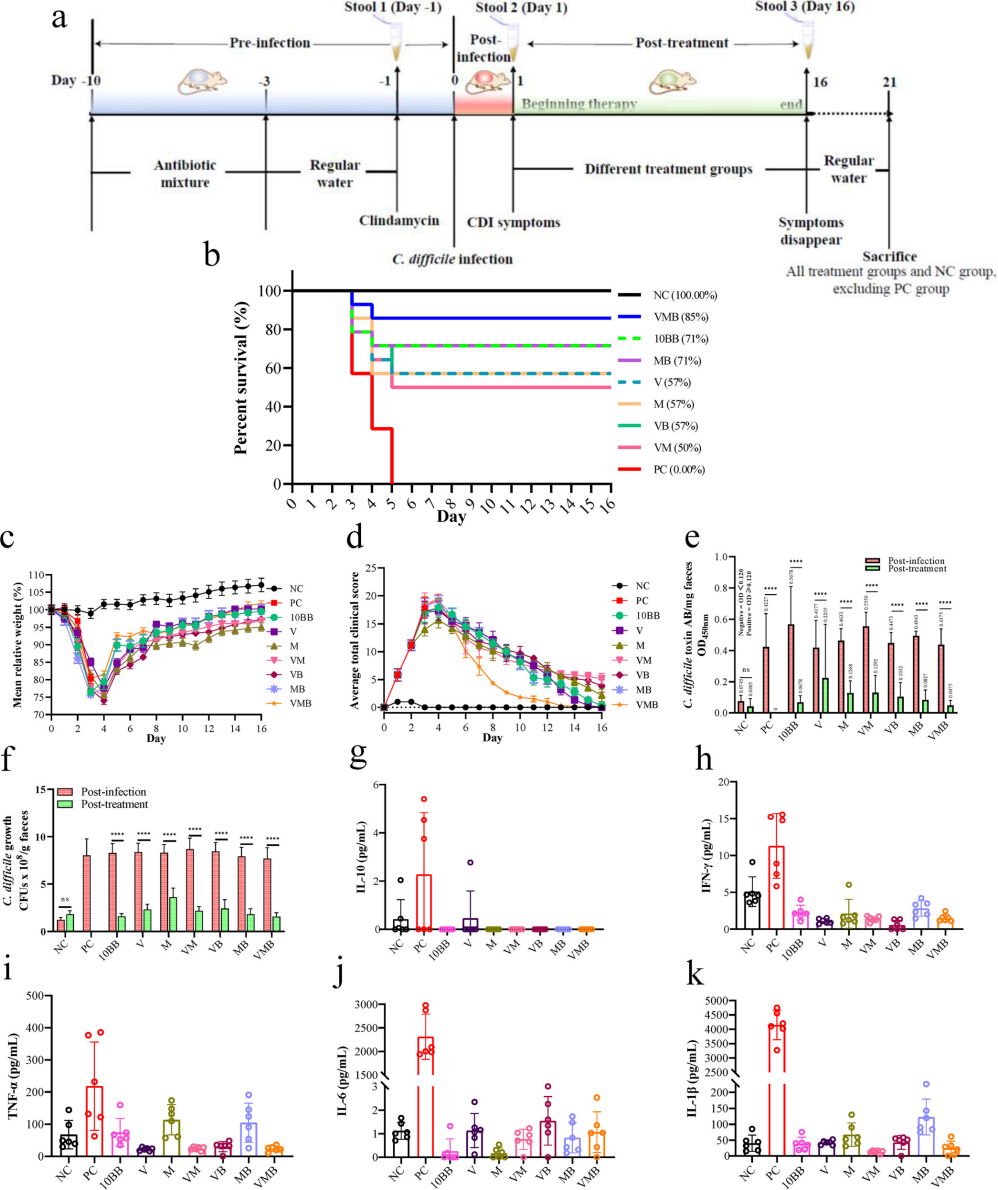
Therapeutic effects of different groups on primary C. difficile infection (pCDI) mice. (a) Experimental design schematics (total *n* = 126). (b) Survival rate (%) on day 16. (c) The mean relative weight (day 0 to day 16). (d) The average total clinical scores (day 0 to day 16). (e) Level of toxin A/B presence in the feces of pCDI mice. OD_450 nm <0_.12 represent negative, OD_450 nm_ ≥0.12 represent positive. The higher the OD value, the higher the toxin content. (f) Isolation of C. difficile from feces of pCDI mice by stool plating in C. difficile moxalactam norfloxacin agar selective medium (CDMN). ns, not significant; ***, *P < *0.0001; Dunnett’s multiple-comparison test. Day 16 to day 21 is the observation period after treatment was discontinued. The levels of (g) IL-10, (h) IFN-γ, (i) TNF-α, (j) IL-6, and (k) IL-1β in the different groups on day 21 after the sacrifice except for the PC group. Once mice in the PC group died, their tissues were immediately collected for measurement. One-way analysis of variance (ANOVA) followed by Tukey’s multiple-comparison test; nonshared letters among different groups represent significant differences; *P < *0.05; *n* = 6 per group. Negative-control group (NC, fed normally without intervention), positive-control group (PC, infected by C. difficile without intervention treatment), 10^10^ CFU/mL of YH68 bacterial suspension (10BB), vancomycin group (V), metronidazole group (M), VAN combined with MTR group (VM), VAN combined with 10BB group (VB), MTR combined with 10BB group (MB), and VAN and MTR combined with 10BB group (VMB).

The experiment consisted of three stages: preinfection (days −10 to 0), postinfection (days 0 to 1), and posttreatment (days 1 to 16). Except for the negative-control (NC) group (100% survival rate), death or moribund state occurred in all other groups, especially in the positive-control (PC) group, where all mice died within 4 days after infection. Day 21 was the experimental endpoint. The final survival rates of pCDI mice after treatment were, in descending order, NC (100%), VAN and MTR combined with 10BB group (VMB; 85%), MTR combined with 10BB group (MB; 71%), 10^10^ CFU/mL of YH68 bacterial suspension (10BB; 71%), vancomycin group (V; 57%), metronidazole group (M; 57%), VAN combined with 10BB group (VB; 57%), VAN combined with MTR group (VM; 50%), and PC (0%) (Fig. 1b). The mean relative weight of mice in the NC group gradually increased over time, while that of mice in the remaining groups decreased significantly within 3 days after infection and did not start to rebound until day 4, and eventually, all groups returned to preinfection levels, except for the M, VM, and VB groups (Fig. 1c). The average clinical scores for all groups peaked within 1 to 5 days after infection and gradually decreased as treatment began, and finally, this value in the V, 10BB, MB, and VMB groups approached zero (Fig. 1d). Notably, the average clinical scores in the VMB group decreased rapidly from the beginning of treatment onwards compared to those of the other groups. The combination of YH68 with antibiotics was able to further reduce the toxin level and colony count of C. difficile in the stool, especially in the MB and VMB groups, which were close to those of the NC group (*P < *0.05) (Fig. 1e and f). The cecum tissues from C. difficile-infected mice exhibited hyperplasia, neutrophil margins, and vascular wall destruction compared to those of uninfected mice, and these clinical signs of disease were relieved or even eradicated with continued treatment (Fig. S1). Interestingly, tissue hyperplasia occurred after antibiotic treatment, while this did not occur in the VB, MB, and VMB groups. The levels of proinflammatory cytokines tumor necrosis factor alpha (TNF-α), interleukin 6 (IL-6), IL-1β, interferon gamma (IFN-γ), and anti-inflammatory cytokine IL-10 in colonic tissue were not significantly different between groups except for the PC group (*P < *0.05) (Fig. 1g to k). Overall, the VMB group displayed an excellent therapeutic effect in pCDI mice, as reflected mainly by the highest survival rate after treatment, significantly lower fecal C. difficile colony counts and toxin levels, and lower colonic tissue cytokine levels.

### Microbial community composition and biomarkers.

**FIG 2:**
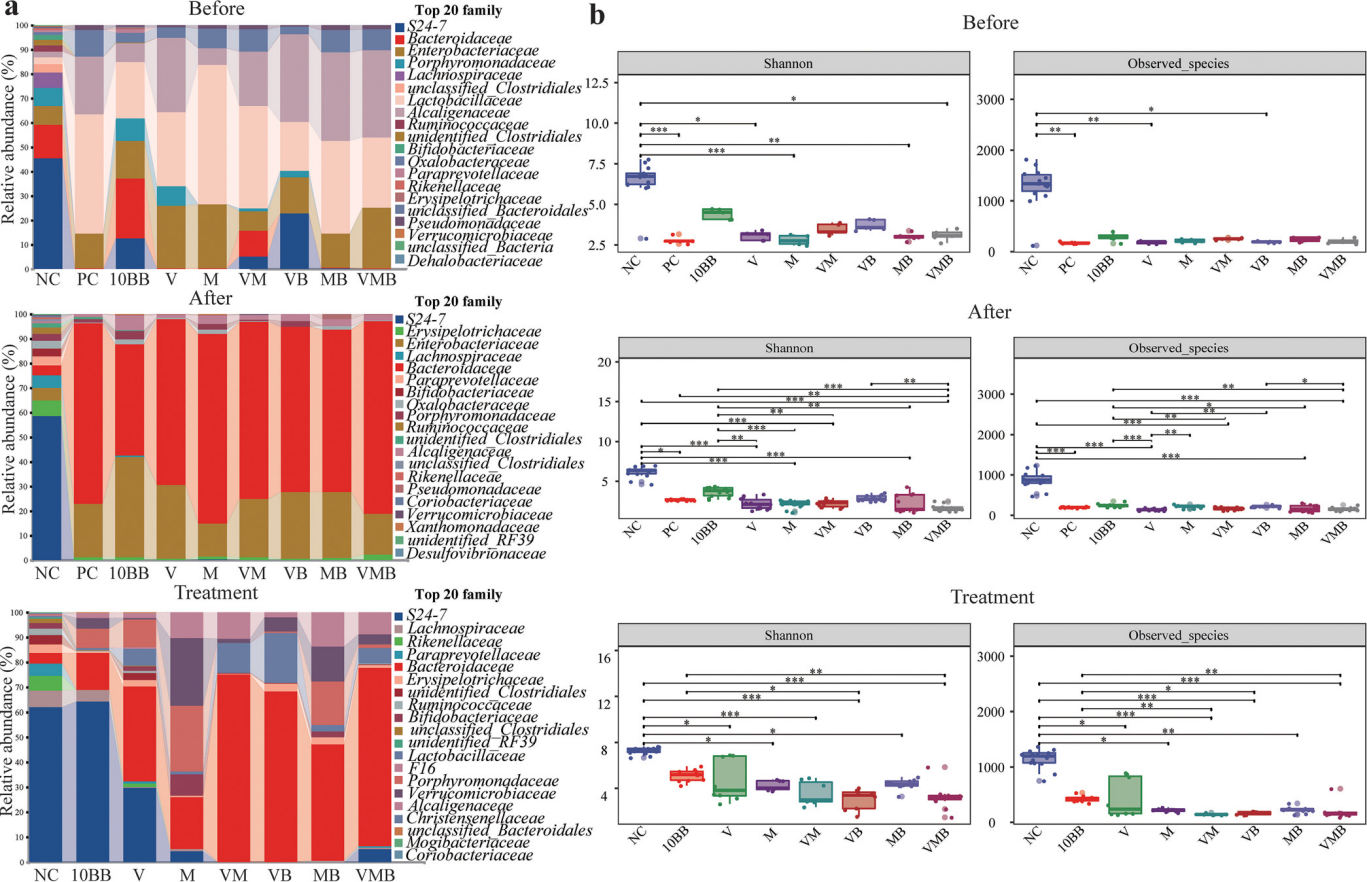
Dynamic changes in the microbial community composition. (a) Microbial community components (family level) at the preinfection, postinfection, and posttreatment stages. Preinfection, NC, *n* = 14; other groups, *n* = 5 per group. Postinfection, all these groups, *n* = 14 per group. Posttreatment, NC, *n* = 14; 10BB, *n* = 10; V, *n* = 8; M, *n* = 8; VM, *n* = 7; VB, *n* = 8; MB, *n* = 10; VMB, *n* = 12. (b) Shannon index and observed species in each group at the three stages. *P* value produced by the Kruskal-Wallis test.

**FIG 3:**
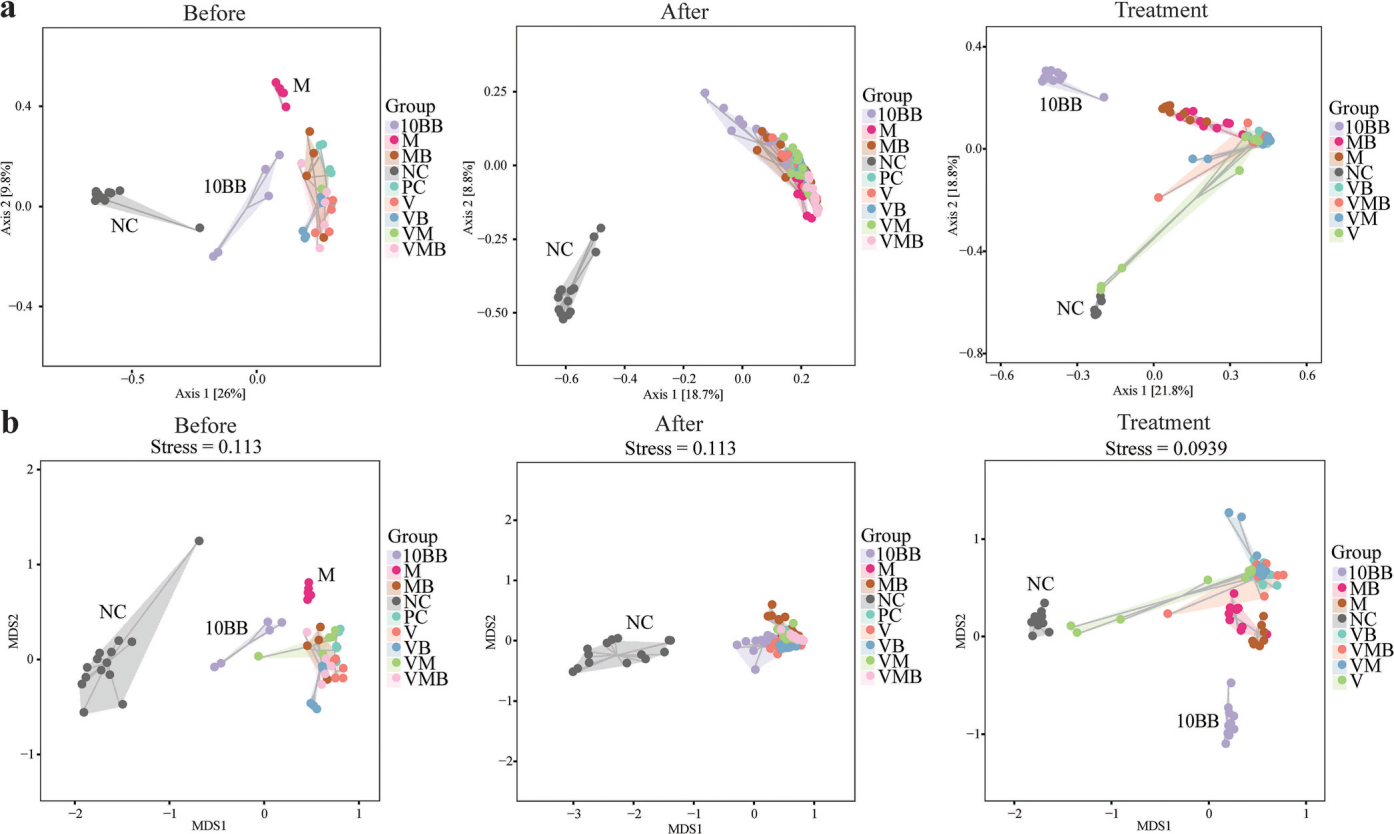
Beta diversity. Each dot in (a) PCoA and (b) NMDS represents a sample, and different-colored dots indicate different groups; the closer the distance between the two points, the smaller the difference between the microbial communities in the two samples.

The total number of raw sequences generated was 14,360,024, the data after removing low-quality sequences was 13,039,993, the effective sequence number after denoising was 12,947,505, and the sequence number after stitching was 12,762,962. After chimeras were removed, the number of high-quality sequences was 11,096,594, the average number per sample was 43,177, and the lowest number per sample was 21,907. The total number of amplicon sequence variants (ASVs) with singleton removed was 11,072,968. We analyzed the fecal microbial structure from pCDI mice at the three stages, focusing on the top 20 families with the highest relative abundance (Fig. 2a). The relative abundances of *S24-7*, *Porphyromonadaceae*, *Lachnospiraceae*, and *Bifidobacteriaceae* were lower in all the groups that were treated with a mixture of antibiotics before infection. The relative abundances of *Bacteroidaceae*, *Enterobacteriaceae*, and *Alcaligenaceae* reached high levels after C. difficile infection, and this trend decreased slightly at the posttreatment stage. After treatment, the difference in the microbial composition of VMB compared to that of VM was reflected mainly in a significantly higher abundance of *S24-7*. Seven-day treatment with a mixture of antibiotics prior to infection resulted in a decrease in alpha diversity levels, and this level decreased further after infection (Fig. 2b). After treatment, the level of alpha diversity rebounded in all groups (especially in 10BB), but no group’s alpha diversity level reached that of the NC group. Notably, the combination of YH68 and antibiotics induced higher alpha diversity levels than the antibiotics used alone (e.g., VMB versus VM). In terms of beta diversity, samples from other groups at the pre- and postinfection stages were separated from the NC group (Fig. 3). After treatment, NC, 10BB, and the remaining groups were separated into three different sections.

**FIG 4:**
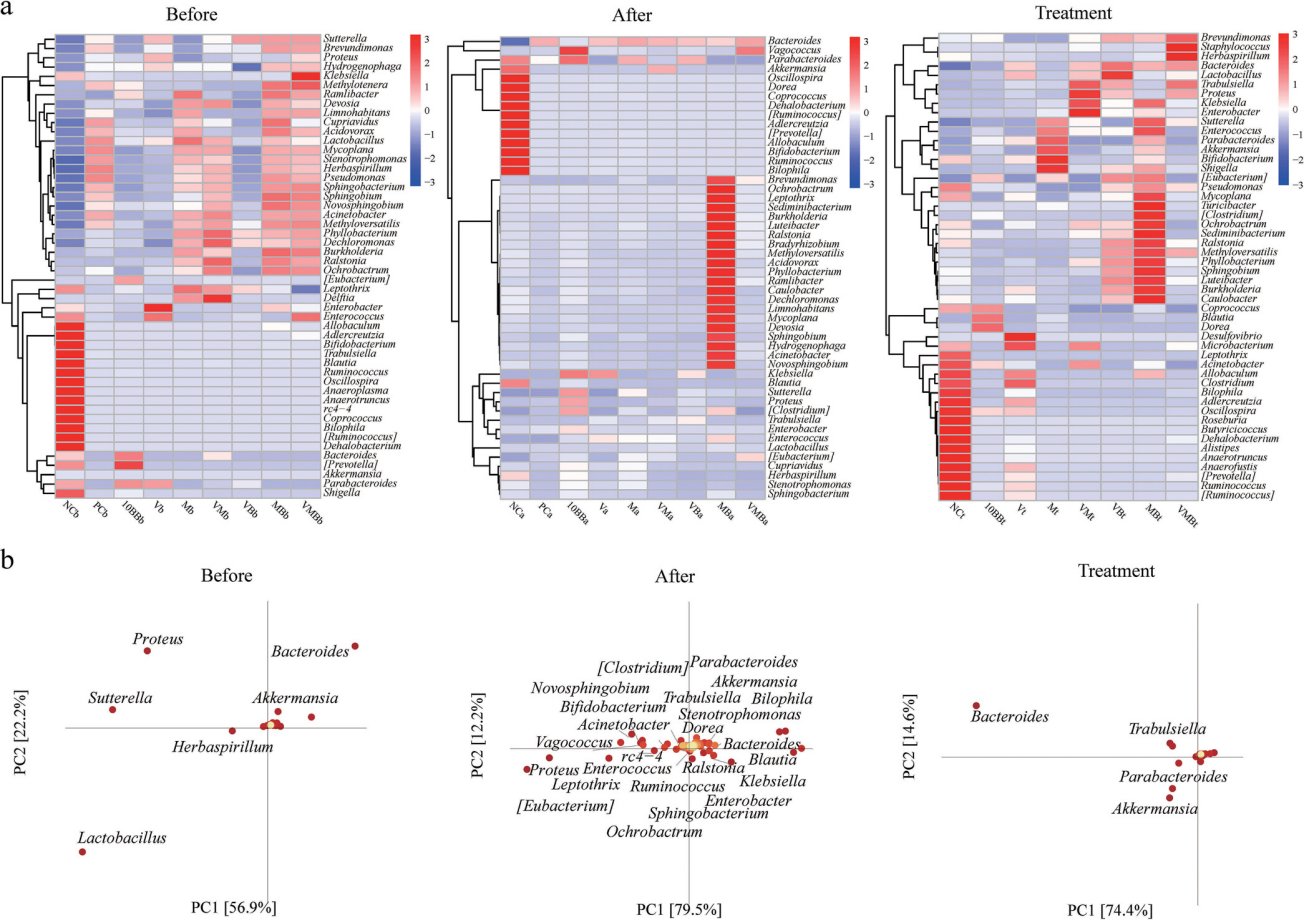
Identification of biomarkers between different groups. (a) Heat map of genus-level microbial composition for species clustering. Sorted by the average abundance of the species in the group; the red color block indicates that the genus is more abundant in the group than the other genera, and the blue color block indicates that the genus is less abundant in the group than the other genera. (b) PCA species load diagram. Each point represents a species at the genus level. The abscissa and ordinate of the point can be considered the contribution of the species to the difference in the two dimensions of the group. The percentages in parentheses on the two coordinate axes are the ratio of the differences in the abundance composition of all samples in this dimension to the total differences. The contribution of the species to the difference in composition between the groups is proportional to the sum of the distances to the coordinate axis. The color from yellow to red indicates its value from small to large. Each point on the right represents a sample. Different-colored points indicate different groups. The closer the projection distance of the two points on the coordinate axis, the more similar the species abundance composition between the two samples in the corresponding dimension.

**FIG 5:**
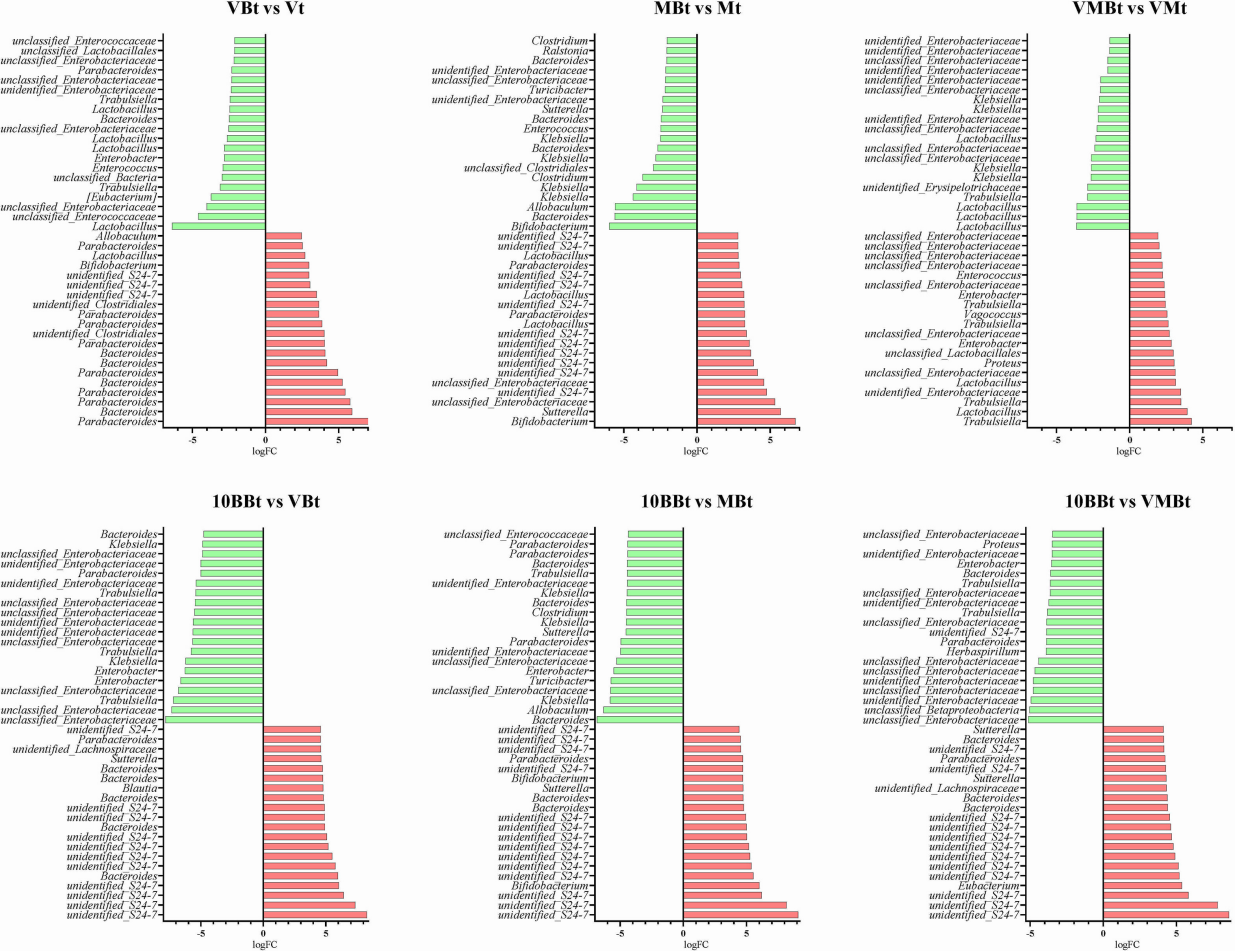
The top 20 genera that were significantly up- and downregulated after treatment. The top 20 genera significantly increased and decreased after treatment in the antibiotic group with the addition of YH68 compared to the nonaddition group and with YH68 used alone compared to the antibiotic group with the addition of YH68.

Differences in microbial community composition (beta diversity) are mainly due to differences in the distribution of specific microorganisms. Thus, the distribution trends and compositional differences in microbial community abundance between samples were further compared and analyzed using heat maps for the top 50 genera in terms of average abundance (Fig. 4a). We found that the *Parabacteroides*, *Akkermansia*, *Oscillospira*, *Coprococcus*, *Bifidobacterium*, *Ruminococcus*, and *Bilophila* genera decreased at the pre- and postinfection stages. Nevertheless, the high abundance of genera varied among the groups after treatment, for example, the high abundance of *Herbaspirillum*, *Brevundimonas*, and *Staphylococcus* in the VMB group. Principal-component analysis (PCA) was used for dimensionality reduction to further analyze the differences in genera abundance composition between groups, and it was found that *Bacteroides* and *Akkermansia* were the shared contributors at the three stages (Fig. 4b). The differences in microbial composition between samples or groups reflect different taxonomic levels. In general, samples or groups with different environmental conditions or large spatiotemporal distribution distances may differ significantly at the phylum and class levels. However, for samples or groups with the same environmental type and similar spatiotemporal distribution, the compositional differences may be limited to the ASV, species, or genus level because there is little difference in taxonomic levels such as phylum and class levels. Therefore, we first attempted to find ASVs where statistically significant differences existed between samples or groups and then determined whether there were trends in the enrichment of these differences at different classification levels. We used the metagenomeSeq method to pairwise compare the groups with the NC group and analyze the ASVs at the order level that was enriched or depleted at three stages (Fig. S2). With the C. difficile invasion and infection, there was an increase of *Bacteroidales* and a decrease of *Sphingomonadales*, *Caulobacterales*, and *Xanthomonadales*. *Verrucomicrobiales* appeared in all groups at the posttreatment stage and showed a high trend of enrichment, while it did not appear in the NC group. Interestingly, the combination of YH68 with antibiotics increased the number and enrichment of certain genus-level communities compared to antibiotics used alone, including *Bifidobacterium*, *Lactobacillus*, and unidentified*_S24-7*, suggesting there was a positive correlation between YH68 and some beneficial microbes (Fig. 5 and Fig. S3). We further compared the microbes in the 10BB group with those in the VB, MB, and VMB groups, and we found an increase in the relative abundance and number of unidentified*_S24-7*, indicating that there was a strong positive correlation between YH68 and unidentified*_S24-7* (Fig. 5).

### Microbial ecology.

**FIG 6:**
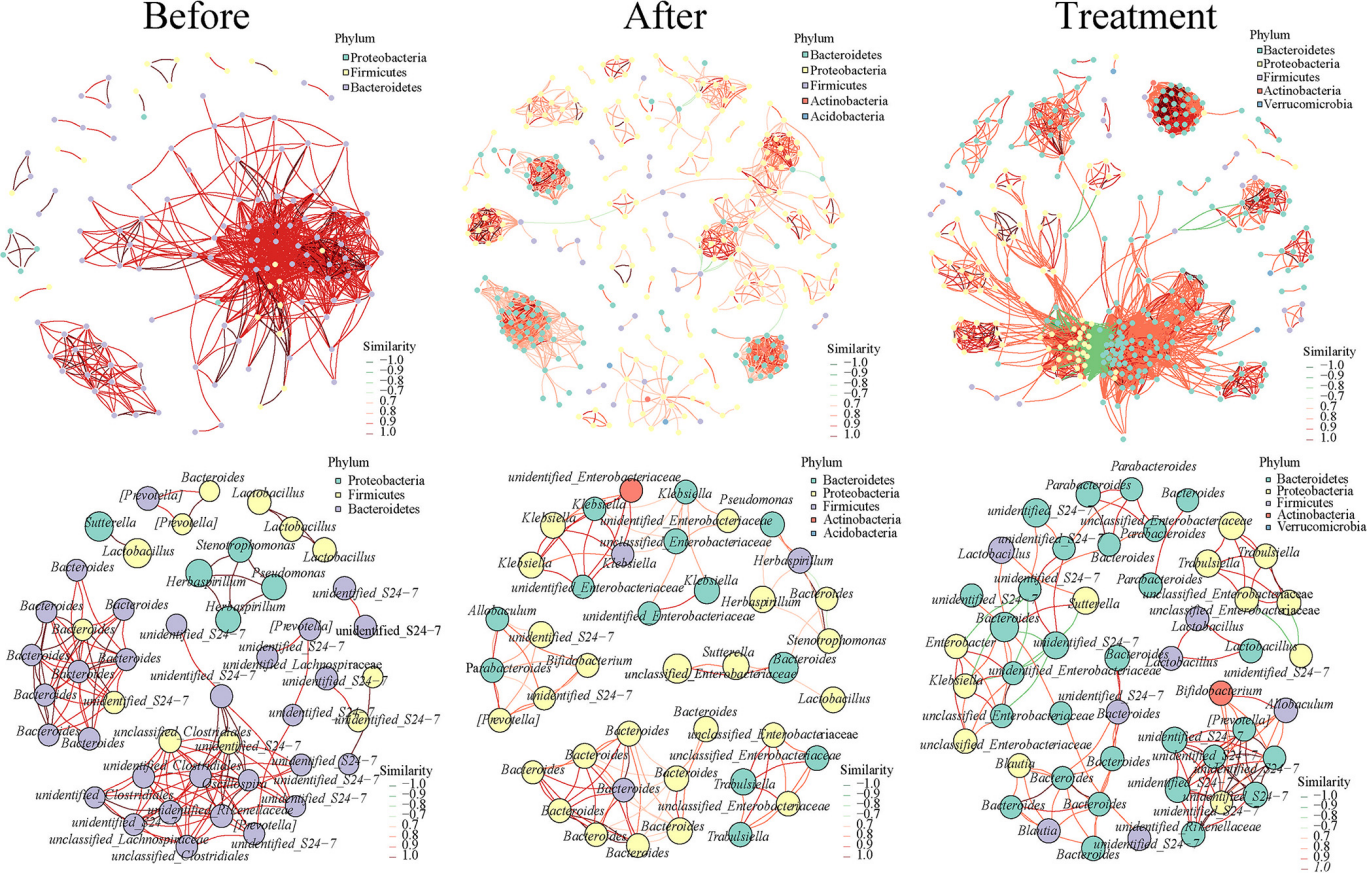
Phylum-level associated network and genus-level annotated dominant seed network at the different stages. The node represents the ASVs in the sample, and the size of the node is proportional to its abundance (in units of log_2_ count-per-millon [CPM/n]); ASVs of the top 50 average abundance in the sample are shown, and the phylum-level and genus-level classification information of the node is marked with different colors; the line between nodes (edge) indicates a correlation between the two nodes being connected, the red line indicates a positive correlation, and the green line indicates a negative correlation. Across the preinfection, the postinfection, and the posttreatment stages, the distribution of dominant genera in the NC group is relatively uniform, whereas the other groups showed large differences, reflected mainly in the single dominant genera at different stages.

The distribution of dominant genera in the NC group was relatively consistent among the three stages, while that in the other groups differed more, mainly in the single dominant genera at different stages (Fig. 6). At the preinfection stage (days −10 to 0), the main phyla were *Bacteroidetes* (first), *Firmicutes* (second), and *Proteobacteria* (third), with a positive correlation between them. *Proteobacteria* rose to first place after infection (days 0 to 1), while *Bacteroidetes* and *Firmicutes* fell to the second and third places, respectively, with a positive correlation, while the *Actinobacteria* and *Acidobacteria* appeared. After completion of treatment (days 1 to 16), the main phyla were *Bacteroidetes* (reverted to the first), *Proteobacteria* (second), *Firmicutes* (third), and *Actinobacteria* (fourth), with the emergence of *Verrucomicrobia*, and there was a significant negative correlation between *Proteobacteria* and *Bacteroidetes*. We further used the seed network map to analyze the top 50 dominant genera (Fig. 6). The relative abundance and number of unidentified*_S24-7* decreased significantly after infection and were accompanied by enrichment of *Bacteroides* and unclassified_*Enterobacteriaceae* (Fig. S4). The number of unidentified*_S24-7* rebounded after treatment and was accompanied by an increase of *Lactobacillus* and *Bifidobacterium*. Overall, 7-day treatment with a mixture of antibiotics prior to infection induced the production of a single dominant genera structure. With the invasion and infection of C. difficile, these groups shared the same dominant genera, including *Enterobacteriaceae*, *Klebsiella*, and *Bacteroides*. The V and M groups shared the same dominant genera, *Parabacteroides* and *Bacteroides*; however, both genera disappeared in the VM group, suggesting that the combination may be detrimental to their growth. It was worth noting that the survival rate of pCDI mice in the VMB group (86%) was higher than that in the VM group (50%), and this process was accompanied by an increase in *Lactobacillus* and a decrease in *Klebsiella*.

### Correlation analysis of fecal microbiota and metabolome.

We selected VMB, 10BB, and MB with excellent therapeutic effects and M and VM with poor therapeutic effects, and we used NC and PC as the controls. Nontargeted metabolomics assays were performed on mice feces of each group at the postinfection (day 1) and posttreatment (day 16) stages. Metabolites with the greatest differences between groups were screened according to the value of orthogonal projections to latent structures discriminant analysis (OPLS-DA) projection variable importance for the projection threshold (variable influence on projection [VIP] of >1). According to the fold change, we further used the first 15 metabolites that were significantly increased and the first 8 metabolites that were significantly decreased after treatment as the main subjects of analysis (Table S1). The metabolites that were significantly upregulated after treatment varied among groups, while shared metabolites emerged among the significantly downregulated metabolites, such as taurocholic acid.

**FIG 7:**
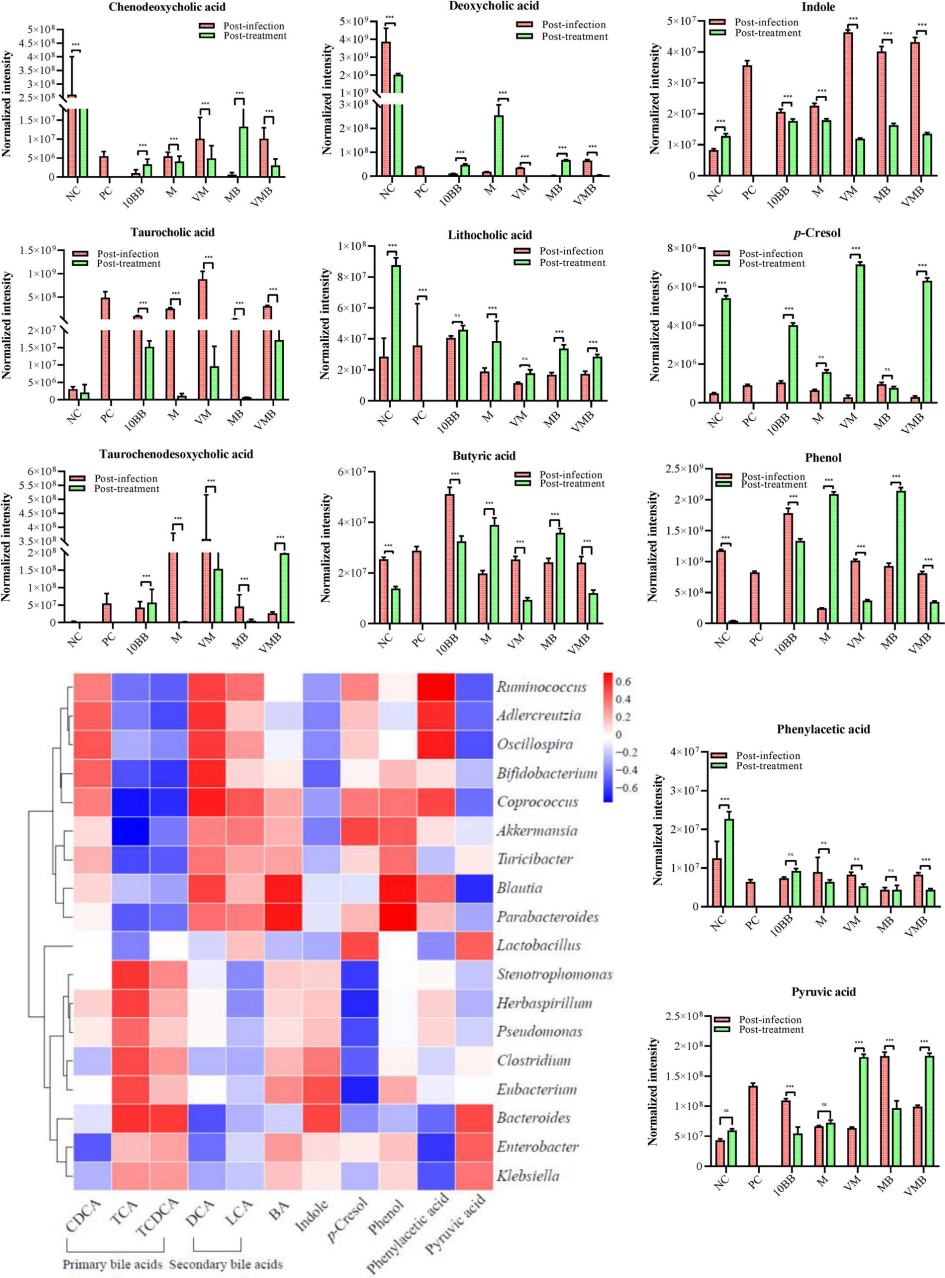
Key metabolite levels and the related microbial community at the postinfection and posttreatment stages. The characteristic metabolites that are closely associated with pCDI include mainly chenodeoxycholic acid (CDCA), taurocholic acid (TCA), taurochenodesoxycholic acid (TCDCA), deoxycholic acid (DCA), lithocholic acid (LCA), pyruvic acid (PA), butyric acid (BA), indole, *p*-cresol, phenol, and phenylacetic acid (PAA). Correlation heat map between these metabolites and microbial community. ns, not significant; ***, *P < *0.001; Dunnett’s multiple-comparisons test.

Considering that no characteristic pattern was shown in each group of significantly up- or downregulated metabolites and that some metabolites may be derived from normal physiological responses, we therefore expanded the range of metabolites closely associated with pCDI, including mainly chenodeoxycholic acid (CDCA), taurocholic acid (TCA), taurochenodesoxycholic acid (TCDCA), deoxycholic acid (DCA), and lithocholic acid (LCA), as well as pyruvic acid (PA), butyric acid (BA), indole, *p*-cresol, phenol, and phenylacetic acid (PAA). CDCA, TCA, and TCDCA are classified as primary bile acids. We found that CDCA decreased in the M, VM, and VMB groups and increased in the 10BB and MB groups after treatment (Fig. 7 and Fig. S6). However, this metabolite was higher in the NC group than in the other groups both before and after treatment, suggesting that the changes of CDCA may be a normal physiological activity. TCA increased substantially after infection and decreased at the posttreatment stage, especially since the levels of TCA in M and MB approached that of the NC group. TCDCA, which elevated after infection, decreased at the posttreatment stage in M, VM, and MB but increased in 10BB and VMB. DCA and LCA are classified as secondary bile acids. After treatment, DCA increased in 10BB, M, and MB, and LCA reached high levels in all groups, with the highest values in M, MB, and VMB. PA increased in all groups after infection, while it decreased in 10BB and MB and increased in VM and VMB at the posttreatment stage. BA decreased in 10BB, VM, and VMB after treatment and increased in M and MB. It is worth noting that BA levels in VM and VMB were comparable to those in the NC group at the posttreatment stage. Indole reached high levels in all groups after infection but decreased in VM, VMB, and MB after treatment (Fig. 7). The levels of *p*-cresol increased (except for MB), while levels of PAA decreased after treatment. Phenol in 10BB, VM, and VMB decreased but increased in M and MB. We further compared and analyzed the differential metabolites between YH68 used alone and YH68 used in combination with antibiotics (Fig. S5 and Table S2). We found that multiple metabolites were significantly upregulated in the combination of YH68 and antibiotics groups compared to the antibiotics used alone groups, with gentamicin C1a being a shared metabolite in MB and VMB. Simultaneously, we found that four of the top five metabolites significantly upregulated in 10BB compared to YH68 used in combination with antibiotics were highly concordant, namely, 2-deoxyadenosine, adenosine, lipoxin A4, and guanosine. It was worth noting that gentamicin C1a, niacinamide, sphinganine, and DCA in VMB were dramatically elevated compared to those in VM at the posttreatment stage.

Correlation analysis of metabolites with microbiome data (genus-level) showed the presence of elevated secondary bile acids in the feces with prominent therapeutic effects in pCDI mice, accompanied by an upregulation of *Oscillospira*, *Bifidobacterium*, *Coprococcus*, and *Akkermansia*, whereas these microbes were negatively correlated with primary bile acids (Fig. 7). PA was negatively correlated with *Blautia*, *Oscillospira*, and *Ruminococcus*. BA was positively correlated with *Blautia* and *Parabacteroides*. Indole was positively correlated with *Clostridium*, while it was negatively correlated with *Ruminococcus*, *Bifidobacterium*, and *Akkermansia*. Phenol and *p*-cresol were positively correlated with *Blautia*, *Akkermansia*, *Parabacteroides*, *Lactobacillus*, and *Coprococcus*. There was a positive correlation between PAA and *Ruminococcus*, *Oscillospira*, and *Adlercreutzia*. 2-Deoxyadenosine, adenosine, lipoxin A4, and guanosine were positively correlated with *Blautia*, *Coprococcus*, *Dorea*, *Oscillospira*, and *Ruminococcus*. Gentamicin C1a was most significantly upregulated in VMB compared to that in VM and was positively correlated with *Akkermansia* and *Coprococcus* (Table S2).

## DISCUSSION

The proven effectiveness of probiotic-related therapies in the prevention of clinical pCDI, including single-dose probiotics and probiotics used in combination with antibiotics (7), implies that probiotics also have the potential to treat pCDI. The results presented in this study revealed that high doses of YH68 used alone or in combination with VAN and MTR exerted prominent therapeutic effects on pCDI mice and did not exhibit side effects. In particular, the VMB group greatly improved the final survival rate of pCDI mice, reduced the levels of colonic inflammatory factors, alleviated cecum lesions, and reduced the number of C. difficile and its toxin protein levels in the feces of mice. Simultaneously, these pCDI mice had higher levels of fecal microbial diversity and key metabolites at the end of treatment. These findings provided a reference for the development and application of probiotic-antibiotic combination therapy for pCDI. Meanwhile, its mode of action needs to be further elucidated.

Many previous reports have focused on the gut microbiota associated with intestinal diseases, including inflammatory bowel disease (IBD), irritable bowel syndrome (IBS), and pCDI (9, 19). Some studies have also shown that high levels of microbial diversity and key metabolites in the gut can form a natural barrier to infection (5). Clinically, CDI patients already showed a significant reduction in diversity prior to antibiotic treatment and significant enrichment of *Enterococcus* and depletion of *Ruminococcus*, *Blautia*, and *Bifidobacterium* compared to non-CDI patients (20), and this was similar to the findings in this study. Therefore, the understanding of the dynamic change pattern of microbiota and metabolome is an important breakthrough point to reveal the mechanism of such intestinal diseases. We found that the microbial structure and the proportion of high-abundance genera composition in the feces of mice in the 10BB group were similar to those in the NC group after treatment, suggesting that the high dose of YH68 itself was able to restore the main microbial communities of pCDI mice to their natural levels. The higher levels of Shannon and observed species index found in the 10BB and MB groups indicated that YH68 and its combination with MTR promoted the reconstruction of main microbial communities in pCDI mice. In terms of the composition of highly abundant genera, the combination of YH68 with antibiotics was able to reduce the destructive effect on the microbiota and indirectly promote the abundance of beneficial genera compared to antibiotics used alone. Shi et al. found that continuous ingestion of ampicillin led to disruption of the gut microbiota, with a decrease in the relative abundance of core genera such as *Lachnospiraceae*, *Coprobacillus*, and *Dorea* and an increase in *Enterococcus* and *Klebsiella* (21). Similarly, the continuous ingestion of mixed antibiotics before infection had already caused damage to the microbiota in this study, thus providing the necessary conditions for C. difficile to easily invade and proliferate. A recent study showed that under antibiotic treatment, C. difficile was able to form symbiotic biofilms with harmful microbes such as *Enterobacteriaceae*, etc., thus enhancing resistance to antibiotics, which may be one of the reasons for the failure in antibiotic treatment of pCDI (22). We found a steady-state microbial community structure and composition in the NC group, whereas the microbiota in pathological processes showed great differences. During infection, if gut microbiota disorders are centered on the arbitrary expansion of various pathogenic genera, mainly C. difficile, are there some common genera that play a central role in the microbiota after YH68 or antibiotic treatment? We found that *Verrucomicrobiales* was highly enriched after treatment (except for VM and V), whereas it did not show an enrichment trend in the contemporaneous NC group, suggesting that this organism emerged after treatment. This result implied that *Verrucomicrobiales* may be a characteristic indicator of healing and recovery in pCDI mice and also suggested that VAN may be detrimental to the growth of *Verrucomicrobiales*. Importantly, *Akkermansia* was the main contributor and key genus among all groups after treatment, and this genus was the only one belonging to *Verrucomicrobiales* (23). This means that changes in the relative abundance of *Akkermansia* may be able to account for the recovery of pCDI mice. *Akkermansia*, especially Akkermansia muciniphila, is closely associated with fatty liver, obesity, diabetes, colitis, and metabolic disorders (24–28) and plays an important role in regulating intestinal mucus thickness and maintaining the integrity of the intestinal barrier (23). Previous studies have shown that intake of *Bifidobacterium* or specific antibiotics increased the proportion of *Akkermansia* in the gut (28), which was similar to the finding in the present study that continued ingestion of YH68 indirectly increased *Akkermansia*. Another characteristic microbe that appeared after treatment was the unidentified*_S24-7*, which was notably enriched only in the 10BB group, suggesting that YH68 can directly affect its abundance. In further microbial association network analysis, the proportion of unidentified*_S24-7* in the top 50 dominant genera decreased after infection but rebounded after treatment. In the same trend, unidentified*_S24-7* was the dominant genus after treatment in both the combination groups (VB, MB, VMB) and the YH68 used alone group (10BB), showing a significant positive correlation with YH68. The unidentified*_S24-7* genus is derived from the *S24-7* genus under the *Bacteroidales* order, and the relative abundance of *S24-7* in the mouse gut is negatively correlated with proinflammatory cytokines, which play an important role in the alleviation of experimental colitis (29). The abundance of unidentified*_S24-7* can also be influenced by other microbes, such as Bifidobacterium longum subsp. *longum* (30). It seems that YH68 in this study stimulated and promoted a significant increase in the relative abundance of *Akkermansia* and *S24-7* in the feces during the treatment of pCDI mice.

Changes in the gut nutritional environment and metabolic profile can markedly affect the activity of the gut microbiota (31). C. difficile alters the gut environment by secreting toxins that induce inflammation to exclude other competitors while obtaining the nutrients needed for its growth and persistent colonization (31, 32). Bile acids play a decisive role in the metabolic activity of C. difficile (33, 34). Primary bile acids include mainly CDCA, TCA, and TCDCA, and secondary bile acids include DCA and LCA, among which secondary bile acids have prominent inhibitory effects on the invasion and colonization of C. difficile (35, 36). We found that dramatically elevated secondary bile acids were positively correlated with *Oscillospira*, *Ruminococcus*, *Bifidobacterium*, *Coprococcus*, *Blautia*, and *Akkermansia*, and previous studies indicated that these microbes secreted a bile acid salt hydrolase that can contain C. difficile spore formation by hydrolyzing primary bile acids, thereby preventing the growth of C. difficile (37, 38). Gut microbes have been shown to ferment carbohydrates and proteins, producing short-chain fatty acids (SCFAs) and an array of metabolites derived from aromatic amino acids (39). SCFAs are important components of intestinal microbial metabolites, including acetate, propionate, and butyrate (40). Acetate is produced by pyruvate through the acetyl coenzyme A (acetyl-CoA) pathway, and pyruvic acid (PA) is an important source for SCFAs production (40). The levels of PA increased after infection but decreased in the 10BB and MB groups at the posttreatment stage. PA was able to be directly taken up and utilized by the C. difficile membrane protein CstA, contributing to its biofilm formation, suggesting that pyruvate-induced biofilm formation may be a key process driving the persistence of C. difficile in the gut (41). Butyric acid (BA) is also one of the important representatives in SCFAs, synthesized from acetyl-CoA or succinyl-CoA (40). We found that BA was positively correlated with *Blautia* and *Parabacteroides*. Aromatic amino acids, such as phenylalanine, tryptophan, and tyrosine, are important sources of phenyl metabolites that can be absorbed by the small intestine or enter the colon directly for excretion in the form of feces (42). PAA is one of the most commonly detected products in healthy human excrement (42). We found that the PAA-associated microbes such as *Oscillospira* were difficult to recover under antibiotic treatment; however, YH68 contributes to the recovery of these microbes. High levels of indole were found in the stools of pCDI patients (37). A previous study has shown that C. difficile induced indole-producing microbes to produce indole and that high levels of indole limited the growth of beneficial indole-sensitive bacteria in the colon and altered colonization resistance, which may allow C. difficile to proliferate and persist (43). We found that indole reached a high level after infection and was positively correlated with *Bacteroides*, *Eubacterium*, and *Clostridium* and negatively correlated with *Bifidobacterium*. This finding was similar to previous studies in which the abundance of intestinal indole-producing bacteria increased and the abundance of beneficial indole-sensitive bacteria such as *Bifidobacterium* decreased with the invasion of C. difficile. C. difficile can ferment tyrosine to produce *p*-cresol, thereby inhibiting the growth of certain intestinal bacteria (44). C. difficile tolerates high concentrations of *p*-cresol better than other microbes, which facilitates the unrestrained growth of C. difficile (45). However, this metabolite was positively correlated with *Akkermansia*, *Lactobacillus*, and *Coprococcus*, suggesting that *p*-cresol may not be a harmful substance derived from C. difficile in the present study. Probiotics are capable of maintaining intestinal homeostasis and host health through a variety of mechanisms in the gut (46). Indeed, the therapeutic effect of YH68 and its combination with antibiotics in pCDI mice may also be an interplay of multiple mechanisms in this study. Prior to infection, antibiotic-induced disturbances in gut ecology and altered nutritional conditions create opportunities for C. difficile growth. On the one hand, C. difficile can meet its own needs by altering its metabolic profile. On the other hand, C. difficile may form symbiotic biofilms to resist antibiotic killing by allying with harmful microbes such as *Enterobacteriaceae*, and key nutrients such as pyruvate play an important role in this process. High doses of YH68 and its combination with antibiotics were able to directly or indirectly upregulate the abundance and number of beneficial microbes in the gut, including *Bifidobacterium*, *Oscillospira*, *Blautia*, and *Akkermansia*. These microbes may inhibit the normal growth of C. difficile by competing with C. difficile for ecological sites and key nutrients. In addition, during the treatment process, the gut inflammatory environment and nutritional conditions also improved. The level of secondary bile acids and BA, which can inhibit the growth and spore germination of C. difficile, increased significantly, while the content of primary bile acids and harmful substances such as indole, which contribute to the growth of C. difficile, decreased. The changes in the inflammatory environment were finally reflected in the restoration of intestinal tissue structure and the downregulation of proinflammatory cytokine levels, the significant reduction of C. difficile toxin content and colony count in the feces of mice, and the high final survival rate. All these results indicated that the treatment of YH68 and its combination with VAN and MTR in pCDI mice was successful. However, it must be admitted that this study leaves much to be desired. First, the fecal microbiome and metabolome information does not accurately reflect the gut microbiome and metabolome information. Second, amplicon sequencing technology does not improve microbiota information to the species level, and nontargeted metabolomics cannot accurately measure changes in each bile acid and short-chain fatty acid. Moreover, although pCDI mice treated with YH68 and its combination with VAN and MTR showed high survival rates, it was worth digging deeper to find out the reasons behind the individual cases of treatment failure. In future studies, it will be necessary to further investigate the mechanism of YH68 in combination with antibiotics in the treatment of pCDI mice and the underlying microbiota changes, the linkage between core microbes and key metabolites, and the influence and interaction of intestinal epithelial cells and mucosal immune function in order to optimize the combination configuration and achieve the best efficacy.

In summary, high doses of YH68 used alone or in combination with VAN and MTR were able to produce significant efficacy and improve ultimate survival in pCDI mice. The combination of YH68 and antibiotic treatment for pCDI mice was achieved mainly by reducing the colony count and toxin levels of fecal C. difficile, reducing the levels of proinflammatory cytokines in intestinal tissues, increasing fecal microbial diversity, reconstructing core genera, and restoring key metabolite levels. This probiotic-antibiotic combination regimen has the potential to be a new option for the clinical treatment of pCDI.

## MATERIALS AND METHODS

### Strains and mice.

Clostridioides difficile ATCC 43255 was purchased from the American Type Culture Collection (ATCC; Manassas, VA, USA), and Bifidobacterium breve YH68 (BB) was purchased from Jiaxing Innocul—Probiotics Co., Ltd. (Jiaxing, Zhejiang, China). C. difficile was cultured in brain heart infusion (BHI) broth, and B. breve YH68 was cultured in de Man Rogosa Sharpe broth supplemented with 0.05% (wt/vol) l-cysteine (MRSC) at 37°C anaerobically (AnaeroGen, Oxoid Ltd., Basingstoke, UK). A total of 126 specific-pathogen-free (SPF) C57BL/6 male mice (6 weeks old) were acquired from Shanghai SLAC Laboratory Animal, Co., Ltd. (Shanghai, China) and were subsequently allowed to acclimatize for 2 weeks before handling. All mice were housed in groups of four per cage at a temperature-controlled (21°C), specific-pathogen-free (SPF) facility and fed a standard chow (SLAC animal Laboratory, Shanghai, China) unless otherwise noted throughout all experiments. All experimental procedures involving mice were approved by the Institutional Animal Care and Use Committee of SLAC (IACUC) Guide for Care and Use of Laboratory Animals (IACUC No. 20190301t0180619).

### C. difficile-infected mouse model.

The mice were infected by C. difficile according to a previously reported method with some modifications (10). The mice were randomly divided into nine groups, including the negative-control group (NC group; *n* = 14), positive-control group (PC group; *n* = 14), 10^10^ CFU per milliliter (10^10^ CFU/mL) of YH68 bacterial suspension group (10BB group; *n* = 14; this dosage was repeated twice a day for 16 days), vancomycin group (V group; *n* = 14), metronidazole group (M group; *n* = 14), VAN combined with MTR group (VM group; *n* = 14), VAN combined with 10BB group (VB; *n* = 14), MTR combined with 10BB group (MB; *n* = 14), and VAN and MTR combined with 10BB group (VMB; *n* = 14). The animal assays were repeated three times independently at different periods, per repeat with the number of mice in each group (2 or 3 mice per cage) being 4, 5, and 5, respectively.

Except for NC, the remaining mice were continuously treated with drinking water containing mixed antibiotics (0.14 mg/mL gentamicin, 1.6 mg/mL kanamycin, 0.86 mg/mL metronidazole, 0.168 mg/mL colistin, and 0.18 mg/mL vancomycin; Macklin, Shanghai, China) for 7 days before receiving one dose of clindamycin (10 mg/kg; Macklin, Shanghai, China), and this stage was called preinfection (days −10 to 0). After 24 h, mice were infected with 3 × 10^8^ CFU of C. difficile by gavage, and all these mice were weighed and monitored daily during the entire experiment (Fig. 1a), with a clinical severity score that varied from 0 (normal) to 20, as described previously (11). This phase within 24 h of C. difficile infection was called postinfection (day 0 to 1). MICs of the different antibiotics used in this study against YH68 were determined by microdilution (17). The results were as follows: kanamycin (>256 μg/mL), gentamicin (96 μg/mL), colistin (<2 μg/mL), metronidazole (<16 μg/mL), vancomycin (<0.25 μg/mL), and clindamycin (<0.016 μg/mL). The NC group was fed normally without intervention, and this group was set up for two purposes, one being that this group was used to compare with other mixed antibiotic-treated groups before C. difficile infection to explore the changes in the gut microbiota induced by mixed antibiotics in different groups and the other one being that the NC group served as a baseline level reference to compare the changes in the gut microbiota in other groups after treatment to examine whether the recovery of the gut microbiota in the treated group was close to the natural level. The PC group was infected by C. difficile without any probiotics or antibiotics treatment. The purpose of the PC group was to observe the pathological process in the infected group under natural progression. For the 10BB group, fresh YH68 bacterial cultures were prepared before gavage to ensure the viability of the strain. Each mouse in the 10BB group was administered 0.2 mL bacterial suspension at 12-h intervals. For the antibiotic groups, including the V (50 mg/kg/day), M (50 mg/kg/day), and VM (50 plus 50 mg/kg/day) groups, antibiotic solutions were prepared in advance and stored at 4°C in the dark. Each mouse in the antibiotic groups was administered 0.2 mL of antibiotic solution at 12-h intervals. For the combination of YH68 and antibiotics groups, including VB, MB, and VMB, each mouse was administered 0.1 mL antibiotic solution for 6 h before 0.1 mL of 10BB bacterial suspension was administered. The time point for treatment initiation was set to be after mice were inoculated with C. difficile and showed significant weight loss, diarrhea, and other common clinical signs of pCDI (24 h). These mice judged to be in a moribund state were euthanized. At the posttreatment stage (day 1 to 16), the treatment was terminated immediately when the treated mice returned to values similar to those before C. difficile infection, including the body weight, the clinical severity score, and a granular stool without any clinical signs of disease (diarrhea). Subsequently, all mice were fed normally and observed for 5 days and then sacrificed.

### Histological analyses and cytokine measurements.

All the mice were sacrificed at the endpoint of the experiment, and their cecum and colon tissues were collected. Once mice in the PC group died or were judged to be in a moribund state and were euthanized, their tissues were immediately collected for pathological analysis. The cecum tissue was flushed with cold 4% paraformaldehyde fixative at 4°C for 24 h and then embedded in paraffin and sliced into 5-μm-thick sections on a microtome (Leica EM UC7, Leica, Munich, Germany). After that, these samples were stained with hematoxylin-eosin (H&E) and observed and photographed using an Olympus microscope (Mod. U-LH100HG, Olympus, Tokyo, Japan). Colon samples (20 mg) from the above mice were homogenized in 400 μL of radioimmunoprecipitation assay (RIPA) buffer (Sigma-Aldrich, Louis, MO, USA) containing protease inhibitors (Santa Cruz Biotechnology, Santa Cruz, CA, USA) and centrifuged at 12,000 × *g* for 10 min at 4°C, and the supernatants were collected. Subsequently, cytometric bead arrays (CBAs) were performed using a CBA mouse Th1/Th2/Th17 cytokine kit (BD Biosciences, NY, USA) with a FACSCanto II flow cytometer (BD Biosciences, NY, USA) to evaluate the concentrations of TNF-α, IL-6, IL-10, IL-1β, and IFN-γ in these supernatants. FCAP Array Software v 3.0 (BD Biosciences, NY, USA) was used to analyze cytometric data.

### Fecal sample collection.

Fecal pellets were collected from mice at three stages, preinfection (days −10 to 0), postinfection (days 0 to 1), and posttreatment (days 1 to 16), respectively (Fig. 1a). The first collection occurred within 24 h before clindamycin injection. The second collection occurred at 24 to 36 h after C. difficile infection. The third collection occurred 24 to 36 h after treatment termination. At each time point, feces from each mouse were collected, quickly placed in sterile Eppendorf (EP) tubes, and stored at −80°C. Fecal samples of each mouse were divided into two portions for 16S rRNA amplicon sequencing and nontargeted metabolomics analysis, respectively.

### Numbers of C. difficile and toxin levels in the fecal sample.

Fecal samples harvested from the different stages were weighed, vortexed in 1.5 mL sterile phosphate-buffered saline (PBS), and kept standing for 15 min. Then, a portion of the fecal supernatant was taken, diluted at 10^−5^ and 10^−6^, and plated on C. difficile moxalactam norfloxacin agar (CDMN; Oxoid, Basingstoke, Hants, UK). Plates were incubated in an anaerobic atmosphere at 37°C for 5 days. The other part of the supernatant was tested for toxin content through a C. difficile TOXA/B II kit (Tech Lab, Kraft Drive Blacksburg, VA, USA).

### 16S rRNA gene amplicon sequencing and bioinformatic analysis.

Total genomic DNA samples were extracted using an OMEGA soil DNA kit (D5625-01; Omega Bio-Tek, Norcross, GA, USA), following the manufacturer’s instructions, and stored at −20°C before further analysis. The quantity and quality of extracted DNA were measured using a NanoDrop ND-1000 spectrophotometer (Thermo Fisher Scientific, Waltham, MA, USA) and agarose gel electrophoresis, respectively. PCR amplification of the bacterial 16S rRNA gene V3-V4 region was performed using the forward primer 338F (5′-ACTCCTACGGGAGGCAGCA-3′) and the reverse primer 806R (5′-GGACTACHVGGGTWTCTAAT-3′). Sample-specific 7-bp barcodes were incorporated into the primers for multiplex sequencing. The PCR components contained 5 μL of buffer (5×), 0.25 μL of Fast *Pfu* DNA polymerase (5 U/μL; Sangon Biotech, Shanghai, China), 2 μL (2.5 mM) of deoxynucleoside triphosphate (dNTP), 1 μL (10 μM) of each forward and reverse primer, 1 μL of DNA template, and 14.75 μL of double-distilled water (ddH_2_O). Thermal cycling consisted of initial denaturation at 98°C for 5 min, followed by 25 cycles consisting of denaturation at 98°C for 30 s, annealing at 53°C for 30 s, and extension at 72°C for 45 s, with a final extension of 5 min at 72°C. PCR amplicons were purified with Vazyme VAHTSTM DNA clean beads (Vazyme, Nanjing, China) and quantified using a Quant-iT PicoGreen dsDNA assay kit (Invitrogen, Carlsbad, CA, USA). After the individual quantification step, amplicons were pooled in equal amounts, and paired-end 2 × 300 bp sequencing was performed using the Illumina MiSeq platform with a MiSeq reagent kit v3 at Shanghai Personal Biotechnology Co., Ltd. (Shanghai, China).

Microbiota bioinformatics was performed with QIIME2 2019.4 (47), with slight modification according to the official tutorials (https://view.qiime2.org/). Briefly, raw sequence data were demultiplexed using the demux plugin followed by primer cutting with the cutadapt plugin (48). Sequences were then quality filtered, denoised, and merged, and chimeras were removed using the DADA2 plugin (49). Nonsingleton amplicon sequence variants (ASVs) were aligned with mafft (50) and used to construct a phylogeny with fasttree2 (51). ASV-level alpha diversity indices, such as the observed species and Shannon diversity index (52), were calculated using the ASV table in QIIME2 and visualized as box plots. ASV-level ranked abundance curves were generated to compare the richness and evenness of ASVs among samples. Beta diversity metrics were estimated using the diversity plugin with samples rarefied to 18,607 sequences per sample. Taxonomy was assigned to ASVs using the classify-sklearn naive Bayes taxonomy classifier in the feature-classifier plugin against the SILVA release 132 database (47). Beta diversity analysis was performed to investigate the structural variation in microbial communities across samples using Bray-Curtis metrics and visualized via principal coordinate analysis (PCoA) and nonmetric multidimensional scaling (NMDS) (53). Principal-component analysis (PCA) was also conducted based on the genus-level compositional profiles (54). Taxon abundances at the ASV levels were statistically compared among samples or groups by metagenomeSeq and visualized as Manhattan plots (55). Random forest analysis was applied to discriminate the samples from different groups using QIIME2 with default settings (56, 57). Cooccurrence network analysis was performed by SparCC analysis. The pseudocount value in SparCC was set to 10^−6^. The cutoff of correlation coefficients was determined to be 70 through random matrix theory-based methods, as implemented in the R package threshold. Based on the correlation coefficients, we constructed a cooccurrence network with nodes representing ASVs and edges representing correlations between these ASVs. The network was visualized using the R packages igraph and ggraph (58, 59).

### Nontargeted metabolomics.

**(i) Metabolite extraction.** One hundred milligrams of fecal pellets (±1%) was added to an Eppendorf (EP) tube (2 mL) containing 2-chlorophenylalanine methanol (–20°C, 4 ppm, 0.6 mL) with a 30-s vortex oscillation. Then, 100 mg of glass beads was added, further ground by a high-throughput tissue grinder for 90 s (60 Hz), and subjected to ultrasound at room temperature for 10 min. Subsequently, the supernatant of the samples was centrifuged (14,000 rpm, 4°C, 10 min) and collected (0.22 μm, sterile filter) for liquid chromatography/mass spectrometry (LC-MS; UltiMate 3000-Q Exactive Focus, Thermo Fisher Scientific, Waltham, MA, USA), and 55 μL of each sample was mixed into a quality control (QC) sample (these QC samples were used to monitor deviations of the analytical results from these pool mixtures and the errors caused by the analytical instrument) (60–62). The rest of the samples were used for LC-MS detection.

**(ii) Chromatographic conditions.** Chromatographic separation was accomplished in a Thermo Ultimate 3000 system equipped with an ACQUITY UPLC HSS T3 (150 by 2.1 mm, 1.8 μm, Waters) column (Thermo Fisher Scientific, Waltham, MA, USA) maintained at 40°C. The temperature of the autosampler was 8°C. Gradient elution of analytes was carried out with 0.1% formic acid in water (solvent C) and 0.1% formic acid in acetonitrile (solvent D) or 5 mM ammonium formate in water (solvent A) and acetonitrile (solvent B) at a flow rate of 0.25 mL/min. Injection of 2 μL of each sample was performed after equilibration. An increasing linear gradient of solvent B (vol/vol) was used as follows: 0 to 1 min, 2% B/D; 1 to 9 min, 2% to 50% B/D; 9 to 12 min, 50% to 98% B/D; 12 to 13.5 min, 98% B/D; 13.5 to 14 min, 98% to 2% B/D; and 14 to 20 min, 2% D, positive model (14 to 17 min, 2% B, negative model).

**(iii) Mass spectrometry conditions.** The electrospray ionization multistage mass spectrometry (ESI-MS*^n^*) experiments were executed on a Thermo Q Exactive Focus mass spectrometer with the spray voltages of 3.8 kV and −2.5 kV in positive and negative modes, respectively. Sheath gas and auxiliary gas were set at 30 and 10 arbitrary units, respectively. The capillary temperature was 325°C. The analyzer scanned over a mass range of *m/z* 81 to 1,000 for full scanning at a mass resolution of 70,000. Data-dependent acquisition (DDA) tandem mass spectrometry (MS/MS) experiments were performed with high-energy collisional dissociation (HCD) scanning. The normalized collision energy was 30 eV. Dynamic exclusion was implemented to remove some unnecessary information in the MS/MS spectra (63).

**(iv) Metabolome data detection.** Based on the base peak chromatogram (BPC), quality control (QC), and quality assurance (QA), it was determined that the dense distribution data of QC samples were reliable. The QC samples were gathered, and the repeatability was good, indicating that the system was stable. In the QC sample, the characteristic peak ratio of relative standard deviation (RSD; <30%) reached approximately 70%, indicating that the data were good. We carried out metabolite hierarchical cluster analysis, sample tree analysis, multivariate statistical analysis, screening, and identification of differential metabolites, and we carried out further statistical analysis and hierarchical cluster analysis of differential metabolism, as well as correlation metabolism heat map analysis and metabolic pathway analysis (63).

### Data availability.

The data that support the findings of this study are available in the manuscript and in the supplemental material of this article. All raw sequences were deposited in the NCBI Sequence Read Archive with submission number SUB7621672. The accession number is PRJNA640496.
