# Supplementary material for: Therapeutic Effects of Bifidobacterium breve YH68 in Combination with Vancomycin and Metronidazole in a Primary Clostridioides difficile-Infected Mouse Model
Source: Microbiol Spectr. 2022 Mar 21;10(2):e00672-22. doi: 10.1128/spectrum.00672-22 (PMC9045379; doi:10.1128/spectrum.00672-22)
Supplement: SUPPLEMENTAL FILE 1 — Supplemental material. Download SPECTRUM00672-22_Supp_1_seq11.pdf, PDF file, 7.4 MB [file spectrum00672-22_supp_1_seq11.pdf]

Therapeutic effects of *Bifidobacterium breve* YH68 in combination with vancomycin and metronidazole in a primary *Clostridioides difficile* infected mouse model

Jingpeng Yang [1*], Lingtong Meng [1], Hong Yang [2*]

[1] School of Food Science and Pharmaceutical Engineering, Nanjing Normal University, 2 Xuelin Road, Qixia District, Nanjing, China

[2] State Key Laboratory of Microbial Metabolism, and School of Life Science & Biotechnology, Shanghai Jiao Tong University, Shanghai, China

*Correspondence:

Jingpeng Yang; e-mail: yang_jp008@163.com; School of Food Science and Pharmaceutical Engineering, Nanjing Normal University, 2 Xuelin Road, Qixia District, Nanjing, China.

Hong Yang; e-mail: hongyang@sjtu.edu.cn; State Key Laboratory of Microbial Metabolism, and School of Life Science & Biotechnology, Shanghai Jiao Tong University, Shanghai, China.

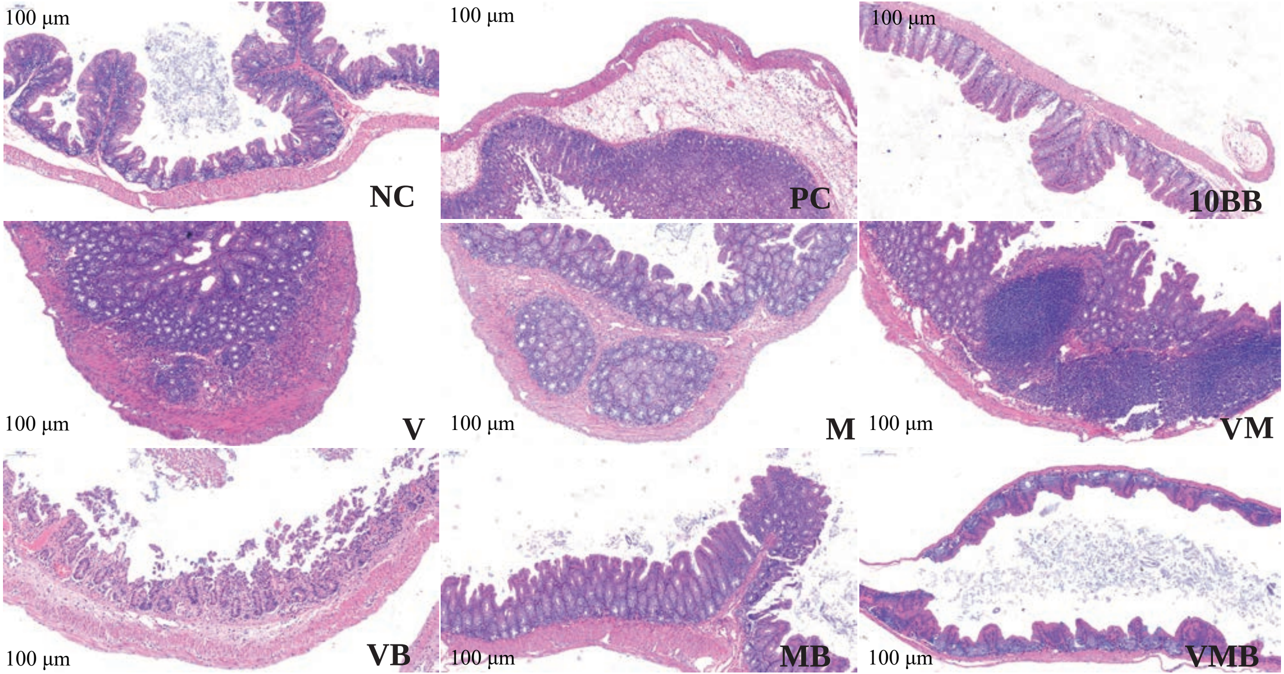

Supplemental Fig. S1 Histopathology of cecum tissues on day 21 after the sacrifice except the PC group. Once mice in the PC group died, their tissues were immediately collected for pathological analysis (n=6 per group; scale bar: 100 µm).

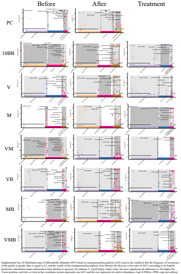

Supplemental Fig. S2 Manhattan map of differentially abundant ASVs based on metagenomeSeq analysis (ASV used as the condition that the frequency of occurrence of the packet is greater than or equal to 0.5, and the results of the metagenomeSeq analysis were filtered; the abscissa is the order of ASVs according to its English taxonomic information name (information from phylum to species); the ordinate is -log10 (adj-p value) value; the more significant the difference is, the higher the Y-axis position; each dot or circle in the coordinate system represents one ASV, and the size represents its relative abundance; log2 (CPM/n), CPM: copy per million; n: sample number; the dotted line separates the significant difference (above) from the insignificant ASV; significantly different points are marked with colored dots or circles, and insignificant ones are indicated with gray circles; significant upward adjustments within this group are displayed with colored solid dots; the color of the dots identifies the horizontal name of the phylum and is marked at the bottom of the figure (the top 10 phyla with significantly increased points); a gray background was added to the first 15 significant points (phylum level), and the name of the purpose is marked at the top; through the above figure, the ASVs in each group that have significantly increased can be found, as well as the phyla and orders that have been increased more).

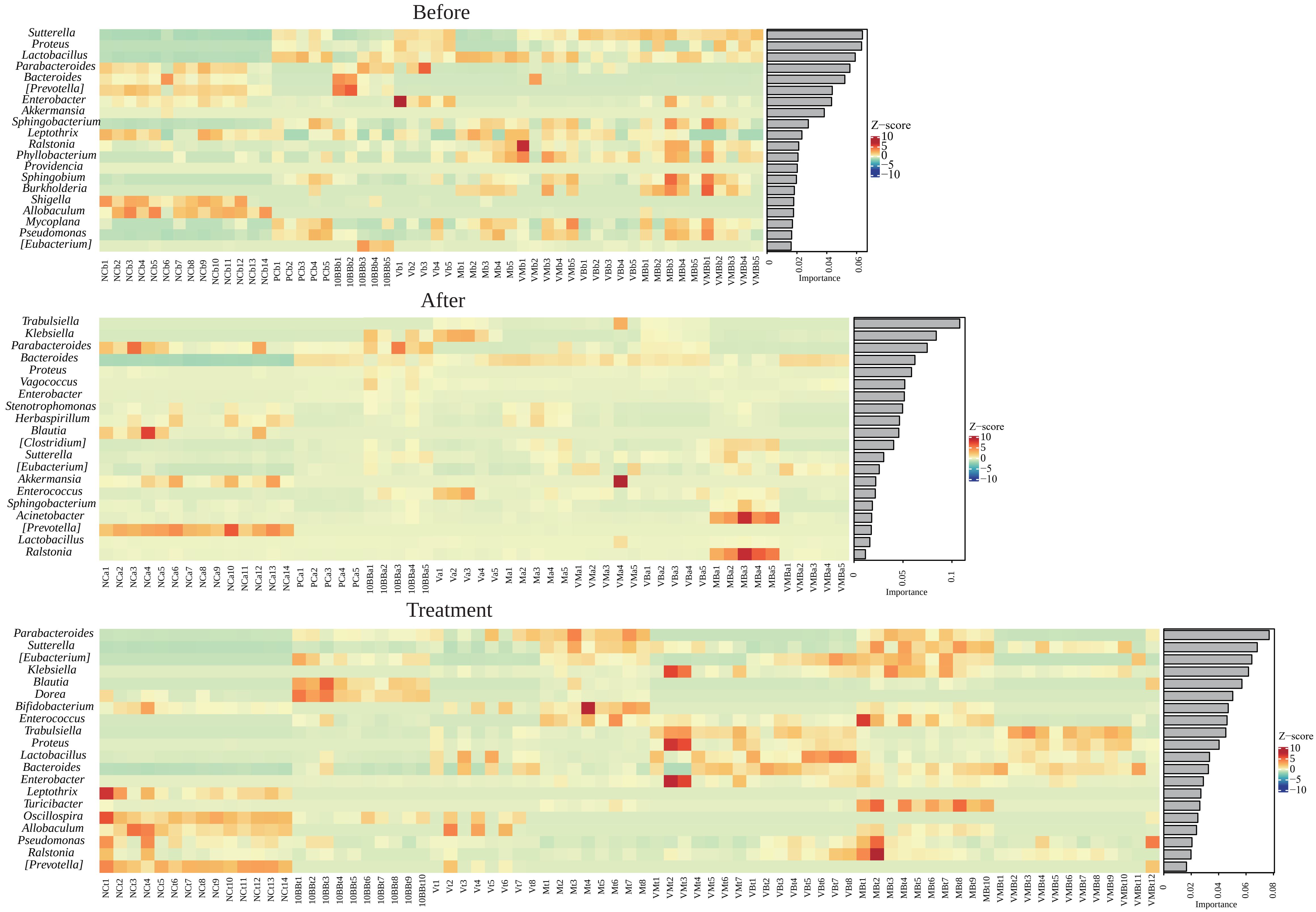

Supplemental Fig. S3 ASV heat map of the top 20 in importance. The abscissa indicates the importance of the species to the classifier model; the ordinate is the ASV name or the name of the taxon at the level of phylum, class, order, family, or genus; from top to bottom, species decrease in the order of impact on their importance of grouping, and these top-ranking species can be considered biomarkers of differences between groups.

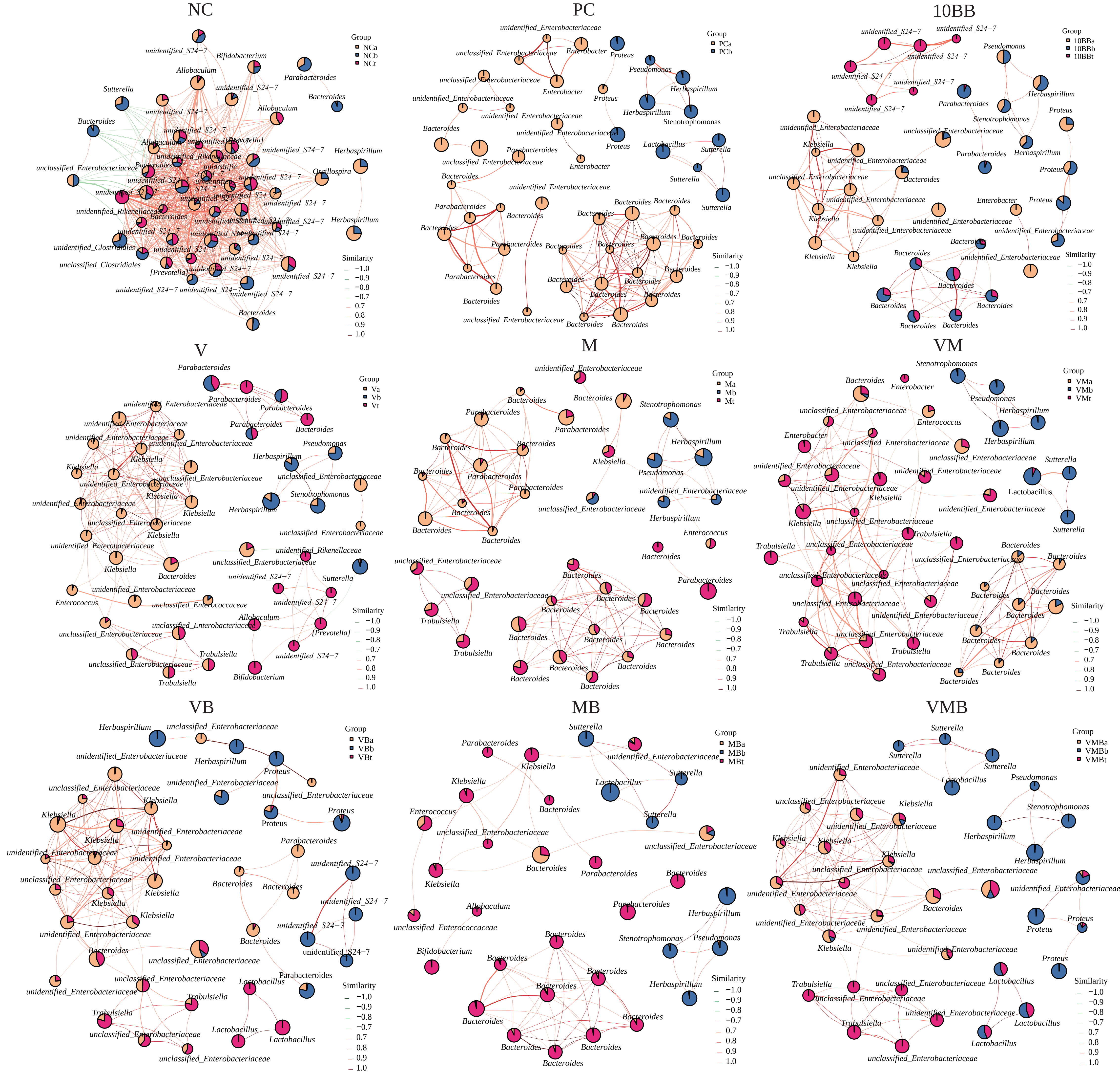

Supplemental Fig. S4 Genus-level annotated dominant seed network at the different stages.

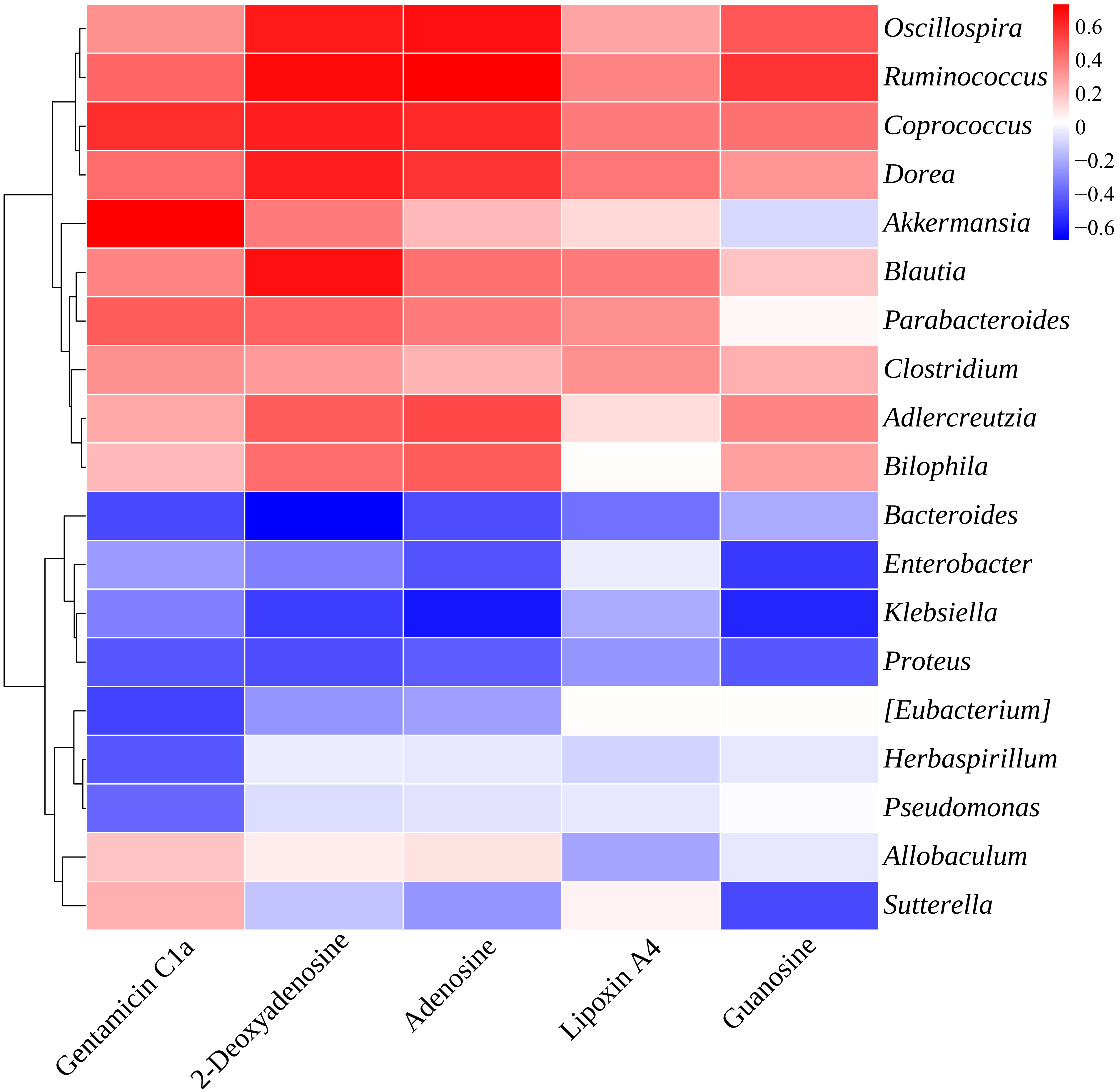

Supplemental Fig. S5 Shared metabolite and related microbial community in the different groups at the post-treatment stage. Shared metabolites were significantly upregulated in the combination of antibiotics and YH68 groups compared to the antibiotic monotherapy group (MBt vs Mt and VMBt vs VMt), and YH68 used alone compared to the combination of antibiotics and YH68 groups (10BBt vs MBt and 10BBt vs VMBt).

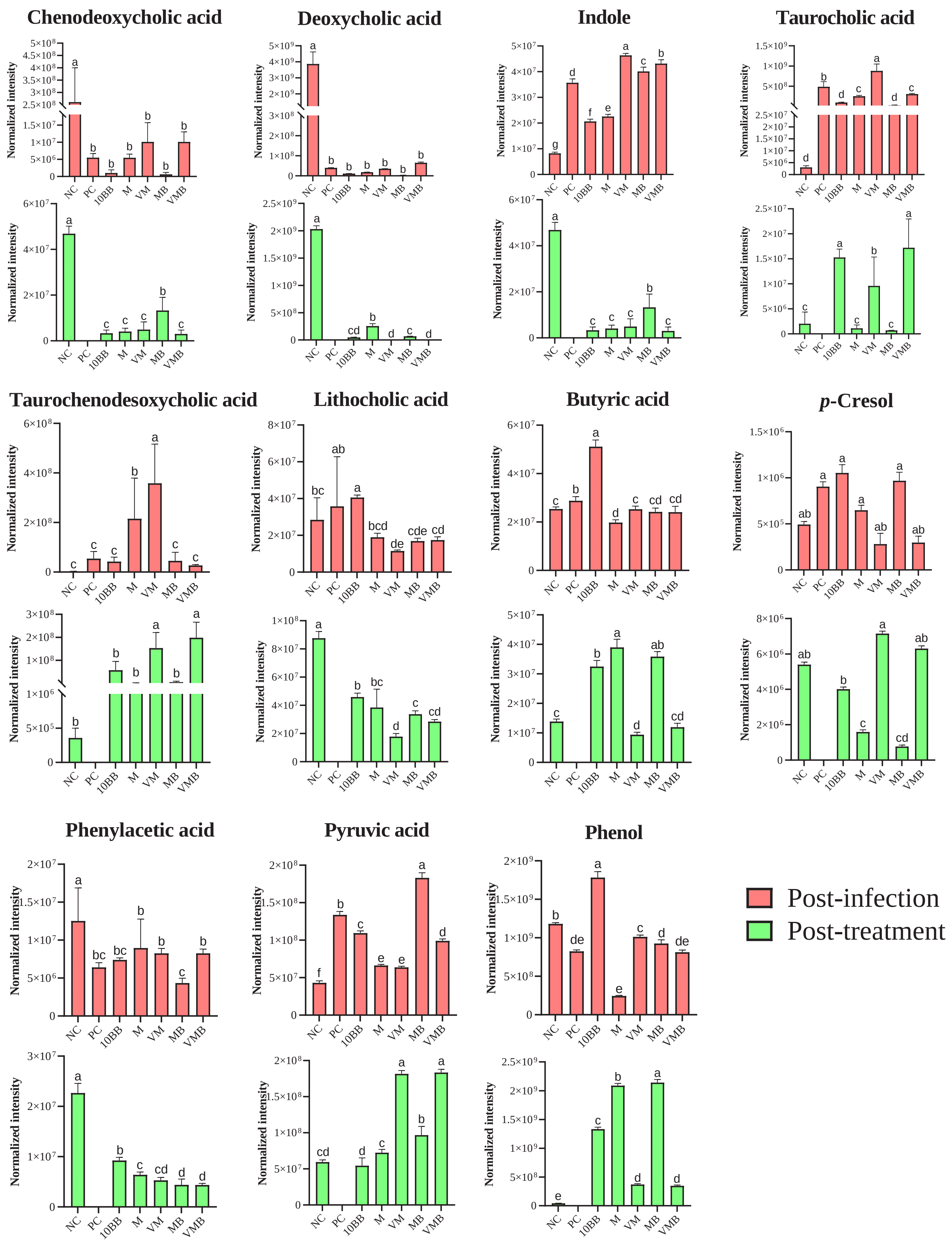

Supplemental Fig. S6 Key metabolite levels at the post-infection "Pink" and post-treatment "Green" stages. One-way analysis of variance [ANOVA] followed by Tukey's multiple comparison test; non-shared letters among different groups at the same stage represents significant differences, $p<0.05$.
